# Supplementary material for: Skeletal Muscle-Specific Bis Depletion Leads to Muscle Dysfunction and Early Death Accompanied by Impairment in Protein Quality Control
Source: Int J Mol Sci. 2023 Jun 1;24(11):9635. doi: 10.3390/ijms24119635 (PMC10253936; doi:10.3390/ijms24119635)
Supplement: Supplementary file 1 [file ijms-24-09635-s001.zip › ijms-2395076-supplementary.pdf]

Table S1. Primers used for qRT-PCR in this study.

Targets	Sequences (5′-3′)	
MYH3	Forward	ACACGGATCAGAGAGCTGGA
	Reverse	CCTTAACACGCCGCTCATAC
myogenin	Forward	CCTTGCTCAGCTCCCTCA
	Reverse	TGGGAGTTGCATTCACTGG
HSPB8	Forward	ATGAAGAGAAGCAGCAGGAAGGTG
	Reverse	TGGTTGTCTTGAGGAAGCTCGTTG
HSPB5	Forward	CGGACTCTCAGAGATGCGTT
	Reverse	TGGGATCCGGTACTTCCTGT
HSP70	Forward	AGACTGTTGAGTTCTTTGTGTTTGGA
	Reverse	GAAGGACCCGACACAAGCAT
CTGF	Forward	CACAGAGTGGAGCGCCTGTTC
	Reverse	GATGCACTTTTTGCCCTTCTTAATG
CYR61	Forward	GATGACCTCCTCGGACTCGAT
	Reverse	GGTTCGGTGCCAAAGACAGG
ANKRD1	Forward	CCTGCGAGGCTGATCTCAAT
	Reverse	GGTCTTCCCAGCACAGTTCTT
GAPDH	Forward	GGTGTGAACGGATTTGGCCGTATT
	Reverse	GGCCTTGACTGTGCCGTTGAATTT

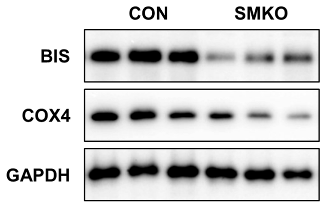

Figure S1. Expression of COX4 in the diaphragm of control and *Bis*-SMKO mice. Expression of BIS, COX4 and GAPDH in the diaphragm of control and *Bis*-SMKO mice was analyzed by western blotting. CON, control; SMKO, *Bis*-SMKO.

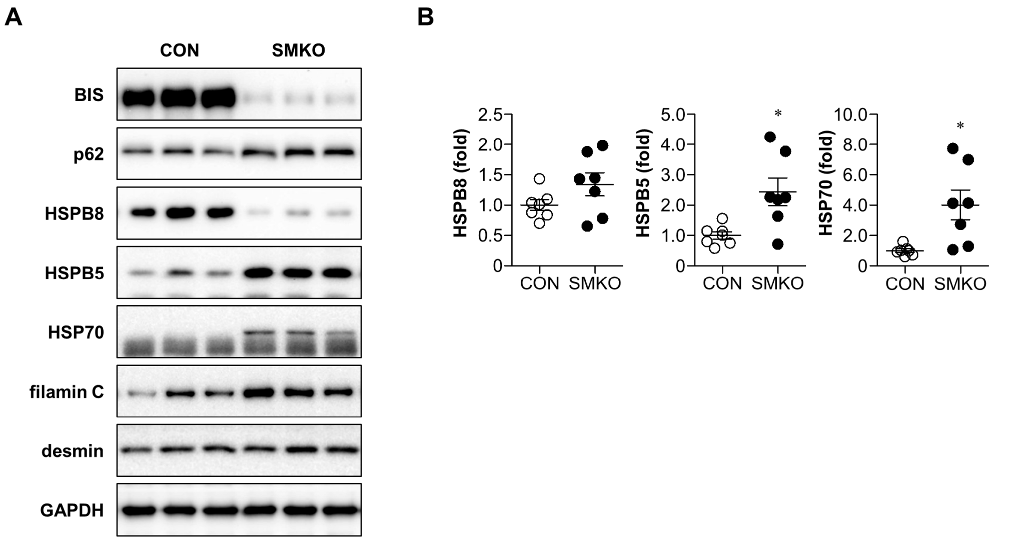

Figure S2. Protein and mRNA expression in the quadriceps of control and *Bis*-SMKO mice. (**A**) Expression of BIS, p62, HSPB8, HSPB5, HSP70, filamin C, desmin and GAPDH in the quadriceps of control and *Bis*-SMKO mice was analyzed by western blotting. (**B**) mRNA expression of HSPB8, HSPB5 and HSP70 in the quadriceps of control and *Bis*-SMKO mice was analyzed by qRT-PCR (N = 7 for both groups) as shown in Figure 2C. CON, control; SMKO, *Bis*-SMKO. Data are presented as mean ± SEM. * $p < 0.05$ compared to control.

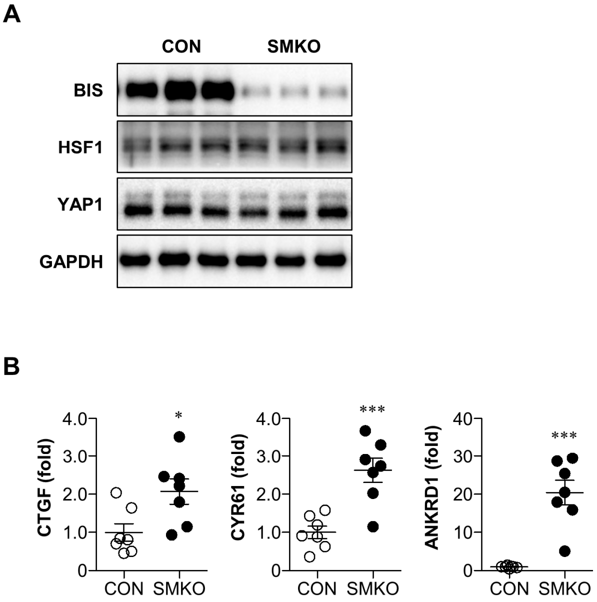

Figure S3. Enhanced YAP1 downstream signaling in the quadriceps of control and *Bis*-SMKO mice. (**A**) Expression of BIS, HSF1, YAP1 and GAPDH in the quadriceps of control and *Bis*-SMKO mice was accessed by western blotting. (**B**) mRNA expression of downstream targets of YAP1 including CTGF, CYR61 and ANKRD1 in the quadriceps of control and *Bis*-SMKO mice was analyzed by qRT-PCR (N = 7 for both groups) as shown in Figure 2C. CON, control; SMKO, *Bis*-SMKO. Data are presented as mean ± SEM. * $p < 0.05$ and *** $p < 0.001$ compared to control.
